# Research on the Frost-Heave Feature of Roadbed Soil Reinforced by Polyurethane Using Distributed Fiber-Optic Sensing

**DOI:** 10.3390/polym17243269

**Published:** 2025-12-09

**Authors:** Jinyong Li, Dingfeng Cao

**Affiliations:** 1 Nanping Wusha Expressway Co., Ltd., Jianyang 354200, China; ljy65099508@163.com; 2 State Key Laboratory for Tunnel Engineering, School of Civil Engineering, Sun Yat-sen University, Zhuhai 519082, China

**Keywords:** optical fiber, distributed monitoring, frozen soil, polyurethane, frost heave

## Abstract

Polyurethane (PU) has proven to be an effective material for reinforcing frozen-soil roadbeds; however, the excessive use of PU increases cost and contamination and limits its large-scale application in practical projects. To fill this gap, laboratory tests were conducted to determine the optimal content that achieved the best reinforcement effect at the lowest cost. A continuous frost-heave strain profile and its variation features were obtained through laboratory tests using advanced Rayleigh optical frequency-domain reflectometry technology (OFDR). A calibration method for OFDR at negative temperatures was introduced. The influences of the PU content, water content, and ambient temperature on frost heave were determined based on distributed measurements. The results indicate that a linear function is suitable for describing the relationship between the strain shift and temperature variation above 0 °C, whereas a cubic function is suggested below 0 °C, with a fitted *R*^2^ of 1. When the moisture content is 4.7% and the ambient temperature is −20 °C, compared with the original reinforced soil, the frost-heave displacement decreased by 33.27%, 47.43%, 71.65%, and 72.77%, respectively, after reinforcement with PU contents of 4%, 8%, 12%, and 16%. When the moisture content increased from 4.7% to 10% and the ambient temperature was −20 °C, compared to the original reinforced soil, the frost-heave displacement of the reinforced soil with PU contents of 4%, 8%, 12%, and 16% increased by 49.34%, 14.93%, 7.48%, and 0.16%, respectively. When the PU content was less than 4%, the reinforcement effect was insignificant. The freezing point and frost heave rate decreased after the addition of PU owing to the changes in the pore structure and matric suction.

## 1. Introduction

Frozen soil, defined as a geomaterial with a temperature below 0 °C, is categorized into three types: short-term frozen soil, seasonal frozen soil, and permafrost (permanently frozen soil) [1]. In the Northern Hemisphere, 50% of the area is frozen ground, of which permanent frozen ground accounts for 21.8% [1]. In China, seasonally frozen areas account for 53.5% of the total land area [2]. In frozen-soil areas, engineering damage caused by freeze–thaw cycles has become one of the most significant natural disasters, such as roadbed damage [2], tunnel lining cracks [3], pipeline bending [3], landsliding [4], and accelerating building material degradation, of which roadbed settlement and deformation are the most common. There are two methods for preventing freezing and thawing deformation: enhancing ground heat extraction and preventing heat intake [5]. The typical methods of enhancing ground heat extraction include the use of crushed rock layers, ventilation ducts, and thermosyphons, while strategies to prevent heat intake involve the application of high-albedo surfaces (or sunshades) and insulation materials [6]. However, these traditional methods have some inherent limitations. For example, high-albedo surfaces and sunshades cannot work at night and are prone to disturbances caused by strong winds. Ventilation ducts bring heat from the ground surface into deep permafrost during summer, thus causing more settlement, and thermosyphons always lead to uneven temperature distributions and settlements [7,8,9]. Therefore, thermal insulation materials such as expanded polystyrene (EPS), extruded polystyrene (XPS), and polyurethane (PU) remain the primary effective measures for reducing frost heave and thaw disasters [10,11]. EPS and XPS layouts are time-consuming, expensive, and disruptive to traffic. Therefore, PU has attracted attention owing to its advantages of low cost, excellent thermal insulating capacity, and high strength [6].

The global annual demand for PU as a raw material has reached USD 66.4 billion, with an average annual growth rate of approximately 7.4%. Zhang et al. [12] proposed that the application of a PU panel could significantly reduce frost heave deformation in high-speed railway subgrades and improve safety and stability. Wang et al. [13] investigated the influence of the injection pressure, dry density, water content, and curing time on the mechanical performance of PU-reinforced soil. Zhou et al. [14] developed a new oil-based PU to reinforce loess embankments and reported that its bond strength was much greater than that of traditional PUs. PU is a versatile material owing to its outstanding performance in terms of high adhesivity, strength, flexibility, and resistance to impact, abrasion, and weather [15]. However, its use in large areas during the process of frozen roadbed treatment is still difficult owing to its high cost. In China, the market price of PU is approximately 4000 USD per ton. One effective measure to reduce costs is to reduce the number of PUs while satisfying subgrade improvement requests. This requires accurate monitoring of the soil frost heave under different PU dosages. Another issue that cannot be ignored is that the use of PU will cause a certain degree of soil pollution. Song et al. [16] found that the iodide diffusivity in the PU-reinforced soil decreased by more than 80%, and the iodide leachability index can exceed the standard value of six, which is set by the U.S. Nuclear Regulatory Commission. From an engineering perspective, while ensuring the effectiveness of PU reinforcement, environmental pollution should be minimized as much as possible. The most effective way to assess the reinforcement effect is to directly monitor the frost heave and thaw settlement before and after reinforcement with PU foundation.

Subgrade settlement monitoring technologies include buried sensors, remote sensing, and ground-based testing methods. Traditional buried sensors include settlement plates, profile settlement tubes, layered settlement rings, point displacement gauges, and strain gauges. However, these sensors are point models. To overcome the shortcomings of traditional sensors, fiber optic sensors (FOSs) have been rapidly developed in recent years owing to their robustness, compactness, and immunity to external electromagnetic interference [17]. FOS can be subdivided into two types based on monitoring features and transmission capabilities: quasi-distributed and fully distributed. Fiber Bragg grating (FBG) is a typical quasi-distributed FOS that has been successfully used to monitor frost heave. Li et al. [3] used an FBG to monitor the frost-heave strain of a secondary lining. Meng et al. [18] attached FBG sensors onto a beam with one end fixed and the other end moving freely, and frost heave was inferred from the beam deflection. Wu et al. [19] investigated the interfacial behavior between the FOS and surrounding frozen soil through a cable pullout test and proposed that the failure model conforms to an elastoplastic model under low-water-content conditions, whereas under high-water-content conditions, it satisfies a soft model. The advantages of FBG technology are its high precision and inexpensive demodulation equipment; however, its disadvantage is that it is expensive if many sensors are used. Remote sensing technologies are suitable for large-scale ground surface monitoring and have difficulty detecting small deformations at the centimeter or even millimeter level, such as the Interferometric Synthetic Aperture Radar (InSAR), and Global Navigation Satellite System (GNSS) [16,20]. Ground-based monitoring methods, such as Ground Penetrating Radar (GPR) and Falling Weight Deflectometer (FWD), are typically applied to detect embankment density, water distribution, voids, and surface settlement [21,22]. However, GPR and FWD are time-consuming and difficult to use in laboratory tests. Therefore, fully distributed OFS technology has great application prospects over large areas and long distances. Among fully distributed OFSs, Rayleigh optical frequency-domain reflectometry (OFDR) is the most suitable technology for assessing subgrade settlement owing to its high accuracy and spatial resolution. OFDR technology has been widely used to monitor the deformation of unfrozen soil; however, its application in frost-heave monitoring has not been reported [23,24,25]. Compared with that in unfrozen soil, the Rayleigh frequency shift caused by temperature variation in frozen soil is greater; therefore, temperature compensation must be performed during the strain monitoring process.

Therefore, the shortcomings of previous research on PU reinforcement of permafrost can be summarized in the following three aspects. First, while the effects of PU on improving soil mechanical strength, porosity, and durability have been established, its effect on mitigating frost heave and spatial strain distribution has not been considered. Therefore, it is necessary to quantitatively determine these effects using OFDR technology. Secondly, the optimal dosage of PU for reinforcing permafrost has not been studied. Insufficient PU content will result in poor reinforcement effect, while excessive dosage will lead to higher economic costs and more environmental pollution. Third, Raman spectroscopy is sensitive to both temperature and strain. Therefore, OFDR technology must be calibrated under negative temperature conditions before it can be used for frozen soil strain monitoring. Previous studies have not yet provided information on this aspect.

To fill the gap, a laboratory test was conducted to evaluate the mechanical performance of PU-reinforced roadbed soil during the freezing process at different ambient temperatures and to determine the influence of PU and water content on the reinforcement effect. Based on this, the optimal dosage of PU was proposed. Frost heave was monitored via OFDR technology, and a calibration method for OFDR at negative temperatures has been proposed.

## 2. Materials and Methods

### 2.1. Theories of Soil Freezing and Strain Monitoring Principle of OFDR

The governing equation of heat transfer for seasonally frozen soil can be expressed as follows [26]:

(1) $${\left(\rho C\right)}_{a}\frac{\partial T}{\partial t}+\rho C_{w}u\cdot \nabla T+\nabla \cdot \left(-\lambda_{a}\nabla T\right)=Q,$$

where ${\left(\rho C\right)}_{a}$ are the equivalent densities and heat capacities at atmospheric pressure, respectively, which can be written as:

(2) $${\left(\rho C\right)}_{eq}=n\left(S_{w}\rho_{w}C_{w}+S_{i}\rho_{i}C_{i}\right)+\left(1-n\right)\rho_{s}C_{s}-n\rho_{i}L_{f}\frac{\partial S_{i}}{\partial T},$$

**Figure 1:**
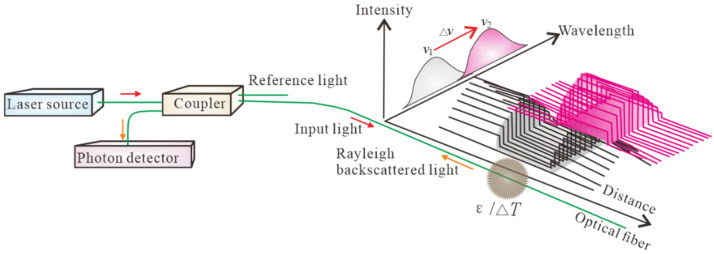
Schematic diagram of the working principle of the OFDR.

The apparent thermal conductivity, $\lambda_{a}$, is [27]:

(3) $$\lambda_{a}=nS_{w}\lambda_{w}+nS_{i}\lambda_{i}+\left(1-n\right)\lambda_{s},$$

where T is soil temperature (°C); n is porosity; $\rho_{s}$, $\rho_{w}$, and $\rho_{i}$ are density of soil, water, and ice, respectively (g/cm^3^); $S_{w}$ and $S_{i}$ represent the saturations of water and ice, respectively, $S_{w}+S_{i}=1$; $C_{s}$, $C_{w}$, and $C_{i}$ are density heat capacities of solid particles, liquid water, and ice, respectively (J/kg·°C); $\lambda_{s}$, $\lambda_{w}$, and $\lambda_{i}$ are thermal conductivities of solid particles, liquid water, and ice, respectively (W/m·°C); $L_{f}$ is the latent heat of phase change (334.56 kJ/kg); u is the fluid velocity field; and Q is the heat source (W/m^3^). Soil frost heave has been suggested to be an anisotropic volume expansion, which can be expressed as follows [28]:

(4) $$\varepsilon =\sqrt[{3}]{1+\eta }-1,$$

where ε and η are the frost-heave strain and ratio, respectively. η is determined by the ice content and can be written as [26]:

(5) $$\eta =\left\{\begin{array}{ll} 0.089\psi_{i}-0.0003 & \psi_{i}\geq 0.003\\ 0 & \psi_{i}<0.003 \end{array}\right.,$$

where $\psi_{i}$ is the ice mass fraction in frozen soil and can be calculated as:

(6) $$\psi_{i}=\frac{nS_{i}\rho_{i}}{\rho_{s}},$$

where Δv is calculated from the Rayleigh frequency shift (Figure 1). The relationship between Δv, *T*, and ε can be expressed as [25,29]:

(7) $$\Delta v=K_{s}\Delta \varepsilon +K_{T}\Delta T,$$

where Δv is the variation in the Rayleigh frequency shift (MHz); $K_{s}$ and $K_{T}$ are the sensitivity coefficients of strain and temperature variations, respectively, assuming that the FOS exhibits the same expansions [30]. Under room temperature conditions (approximately 25 °C), the assumption is correct; therefore, $K_{s}$ and $K_{T}$ are constants, which has been widely proven [25,29]. The $K_{s}$ and $K_{T}$ below 0 °C have not been calibrated.

### 2.2. Sensor Calibration

**Figure 2:**
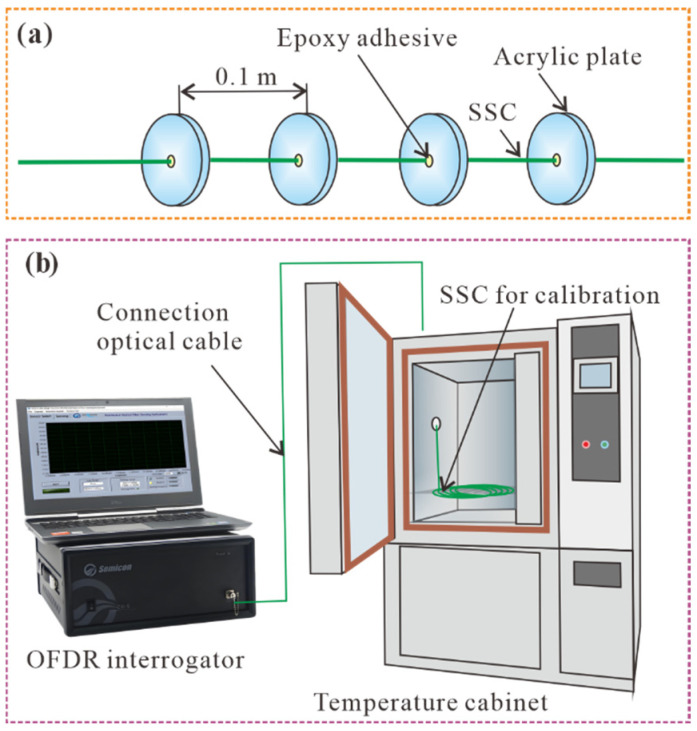
Schematic diagram of the sensors and calibration device: (**a**) strain-sensing cable (SSC) with clamps and (**b**) OFDR interrogator and temperature cabinet for temperature compensation.

When optical cables are used to monitor soil deformation in laboratory, some scholars have suggested installing an acrylic plate to prevent slippage between the strain-sensing cable (SSC) and surrounding soil and increase its coordination with soil deformation [31,32,33]. Therefore, a 2 mm diameter polyurethane sheathed SSC was used. The acrylic plate was made of an acrylic board with a diameter of 20 mm and a thickness of 2 mm. A hole with a diameter of 2 mm was drilled at the center of the plate. The SSC was fixed to the inner hole of the plate by using epoxy glue, as shown in Figure 2a. The distance between adjacent plates was 0.1 m. A 10-meter-long SSC was placed in a temperature cabinet that was kept loose before calibration, as shown in Figure 2b. The calibration temperature was divided into 11 levels: 20, 15, 5, 0, −5, −10, −15, −20, −25, and −30 °C. When each calibration temperature level was set, the strain of the optical fiber in the temperature cabinet was measured using the OFDR after the ambient temperature stabilized. The criterion for judging the temperature stability is that the strain of two consecutive acquisitions is less than 5 με. The time interval between the two adjacent samplings was 10 s. The average value of ten sets of data collected after stabilization was selected as the strain drift at this temperature level.

### 2.3. Experimental Materials and Procedures

**Figure 3:**
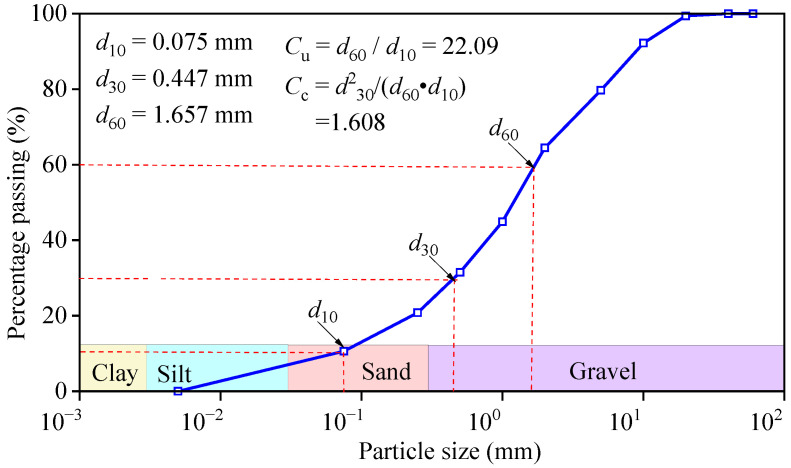
Particle size distribution.

Soil from the high-altitude region of Golmud City, Qinghai Province, was used as the test soil. The basic physical parameters of the soil samples were measured before the experiment, as shown in Figure 3 and Table 1. The particle size distribution of the soil samples was determined using the densitometer method and the sieve analysis method. The liquid and plastic limits of moisture content are 39.1% and 25.2%. The comical composition was measured by X-ray Fluorescence Spectrometer (XRF).

**Figure 4:**
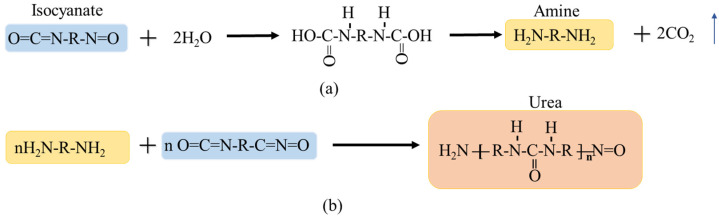
Basic chemical principles of PU-reinforced soil: (**a**) amine production reaction and (**b**) urea production reaction.

In polyurethane (PU) in this study, isocyanate is the main reactant, along with catalysts, foaming agents, and other auxiliary agents, to form a cured body through multi-stage polymerization and cross-linking reactions. The type and amount of main and auxiliary agents have a significant impact on the physical and mechanical properties of the resulting polymer. Isocyanate molecules are compounds containing an isocyanate group (-NCO). The isocyanate group is chemically reactive and readily reacts with hydroxyl groups, water, and amines. The isocyanate group reacts with amino groups to form ureas, which constitute the hard structure of polymers, and reacts with urethane groups to form urethane esters, which also improve the mechanical properties of polymers. Therefore, appropriately increasing the amount of isocyanate can improve the strength of polymers. The isocyanate group (-NCO) reacts with water to produce amines and carbon dioxide gas, as shown in Figure 4. As the reaction progresses, the isocyanate groups react with the amino groups generated during the reaction and those in the catalyst to form urea, which forms the rigid structure of the polymer.

**Figure 5:**
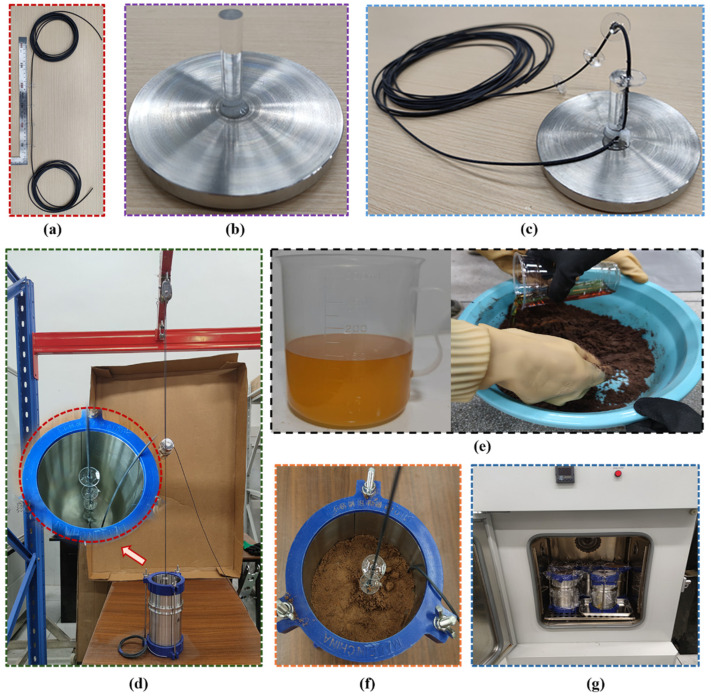
Experimental procedures: (**a**) acrylic plates installed on SSC, (**b**) an acrylic column fixed to the bottom of the ring knife, (**c**) SSC attached to the acrylic column, (**d**) SSC pretensioned with a 1 kg weight, (**e**) polyurethane and soil evenly mixed, (**f**) reinforced soil filling, and (**g**) ring-knife samples placed into the temperature cabinet.

The experimental procedure is illustrated in Figure 5. The SSC with four acrylic plates was the same as that used for the calibration in Figure 2. A custom-made ring knife with a height of 200 mm and an inner diameter of 100 mm was used to pack the soil. The sidewall of the ring knife was divided into two pieces and fixed using a metal hoop. The sidewalls and base were fixed using three screws. A 40 mm long acrylic rod with a diameter of 10 mm was fixed to the base to fix the SSC, as shown in Figure 5b,c. Because the SSC in a relaxed state cannot deform synchronously with the surrounding soil, it must be pretensioned before being used to monitor soil deformation [32]. Here, the SSC fixed on the base was pre-tensioned to 10,000 με using a weight and fixed pulley, as shown in Figure 5d. Liquid PU was produced by Wanhua Chemical Group Co., Ltd., Dalian, China, and its primary chemical composition was isocyanate and surfactant. Isocyanates can react with water in the soil, yielding unstable carbamic acid, which gradually decomposes to form amines and carbon dioxide, as shown in Figure 5a. The resulting amines can further react with isocyanate to yield urea with high strength, as shown in Figure 5b. Figure 5e,f shows the processes of mixing PU with the soil and filling the ring-knife column, respectively. The inner wall was coated with lubricating oil and covered with thin plastic wrap to reduce surface friction so that the soil sample could freely expand in the vertical direction after freezing.

Since PU undergoes an immediate chemical reaction upon contact with water, the reaction rate is related to the catalyst used. In this study, the time from the start of the water reaction to complete curing was 60 min. A method of mixing and layering was adopted. Each soil sample weighed 200 g, and the corresponding amount of PU for the test was measured and poured in drop by drop using a graduated cylinder. During the pouring process, the mixture was stirred in a glass to ensure that the PU and soil were evenly mixed. A sign that the soil and PU are mixed evenly is that there are no binders with a diameter greater than 5 mm after the PU and soil sample are mixed. After mixing, the soil with PU added evenly is poured into the mold and compacted continuously using a PVC pipe with a plug. After filling was completed, the surface was covered with a layer of insurance film to prevent water evaporation, and a ring knife was placed in the temperature cabinet, as shown in Figure 5g. Before cooling, the soil sample was placed at room temperature for 24 h to allow the PU and moisture to fully react chemically.

The experimental procedure is divided into two stages. During the first stage, soil with a natural gravimetric water content of 4.7% was collected from Golmud (Qinghai, China). Five soil samples with PU mass percentages of 0%, 4%, 8%, 12%, and 16% were placed in a temperature cabinet for 48 h at a constant temperature of 20 °C to allow the PU to fully react with the water in the soil. Thereafter, the inner temperature was decreased to 0, −1, −3, −5, 10, −10, −15, and −20 °C. The temperature was then maintained for 8 h. During the entire temperature decrease process, the strain was measured every 10 min. After all these measurements were completed, the experimental process entered the second stage, in which additional samples were added to increase the water content to 10%. The newly added water was slowly dripped onto the top via a dropper. After drying, the samples were allowed to equilibrate for 48 h to allow water to penetrate deeper into the soil under the action of gravity and capillary forces and to reach a stable state.

## 3. Results and Discussion

### 3.1. Temperature Compensation Calibrations of SSC

**Figure 6:**
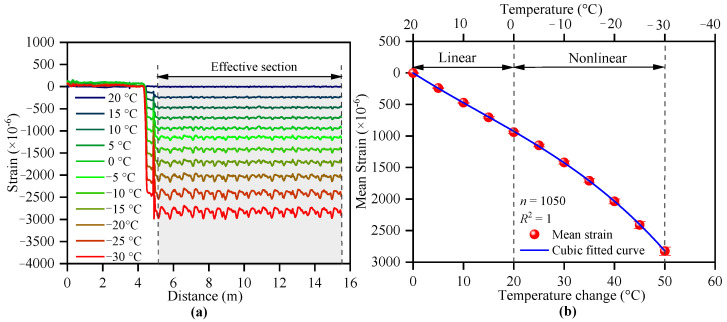
Temperature calibration results of an unstressed SSC: (**a**) strain shift caused by temperature and (**b**) relationship between strain shift and temperature variation.

The temperature calibration measurement results are shown in Figure 6. The total SSC length of 15.7 m can be divided into three sections: the outside section, where the strain does not change; the section passing through the temperature cabinet wall, where the strain gradually changes; and the section fully inside the cabinet, where the strain changes steadily, as shown in Figure 6a. A fully inside section is an effective section that can be used for calibration. Figure 6a also shows slight fluctuations in the effective section caused by uneven temperatures. The SSC was placed on a metal wire rack; therefore, the temperature of the contact area between the SSC and the rack was lower owing to heat conduction, whereas the temperature of the remaining parts was completely determined by the air temperature in the temperature cabinet. The average temperature data collected from 1050 positions in the effective section were calculated to reduce the influence of fluctuations. The relationship between the temperature change and the mean strain shift is shown in Figure 6b. The strain-temperature relation curve is linear above 0 °C and nonlinear below 0 °C. The lower the temperature is, the more evident the nonlinearity becomes, indicating that $K_{s}$ in Equation (7) is not constant. There is currently no rigorous mathematical expression for temperature and the resulting strain drift; in general, empirical models are used for fitting. Here, a cubic function was used to describe the strain shift caused by temperature change, as shown in Figure 6. A fitted curve with *R*^2^ = 1 is shown in Figure 5b. The fitted parameters are listed in Table 2.

(8) $$\Delta \varepsilon_{T}=A+B\Delta T+C\Delta T^{2}+D\Delta T^{3},$$

where $\Delta \varepsilon_{T}$ is the strain caused by temperature. *A*, *B*, *C,* and *D* are parameters fitted using the least squares method.

### 3.2. Frost Heave of PU-Reinforced Soil with Different Contents of PU

**Figure 7:**
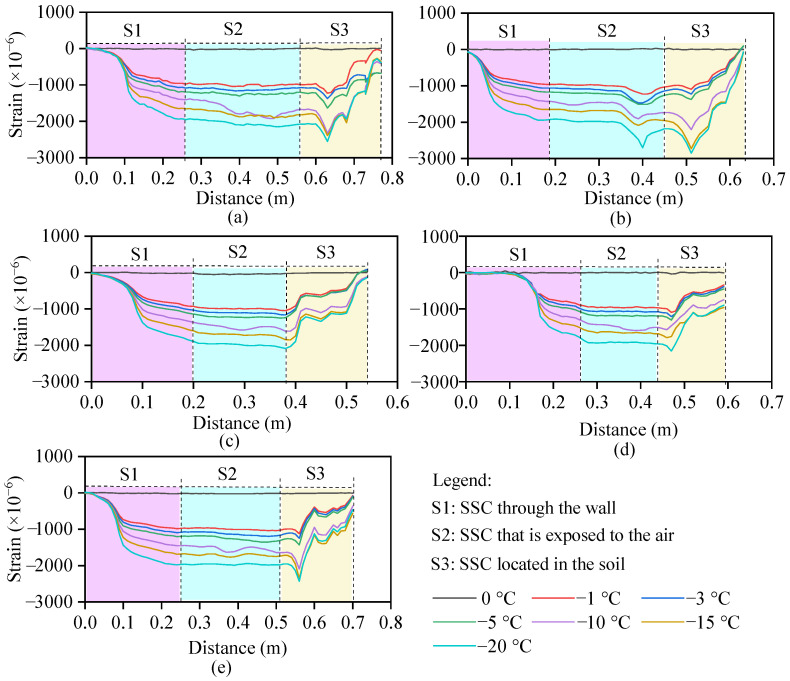
Frost-heave strain development during the freezing process of soil with different contents of PU at a 4.7% water content: (**a**) PU = 0, (**b**) PU = 4%, (**c**) PU = 8%, (**d**) PU = 12%, and (**e**) PU = 16%.

The frost-heave strain development during the freezing process of soils with different PU contents and 5% water contents is shown in Figure 7. Three sections can be observed along the SSC distance: the cable passing through the wall of the temperature cabinet (S1), the section exposed to air in the temperature cabinet (S2), and the section buried in the soil (S3). The strain changes in S1 and S2 were caused completely by the Rayleigh frequency drift caused by the temperature drop, whereas that in S3 was affected by both the temperature decrease and soil frost heave. Compared with S2, S3 had a smaller strain, and the difference between S2 and S3 was the frost-heave strain. The strain curve shows seven steps, corresponding to seven levels of ambient temperature: 0, −1, −3, −5, −10, −15, and −20 °C.

**Figure 8:**
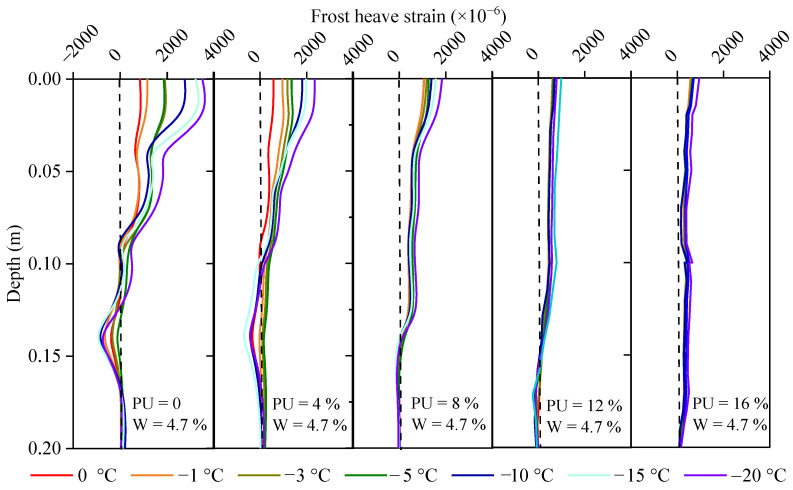
Stain profiles of the soils with different PU contents at a water content of 4.7%.

The frost-heave strain profiles calculated from Figure 6 after temperature compensation are shown in Figure 8. Figure 8 shows that for the soil samples with PU contents of 0, 4, and 8%, the frost-heave strains decrease with depth for two reasons. The final strain is controlled by both the soil pressure caused by gravity and the frost-heave force. The greater the depth is, the greater the soil pressure, and the smaller the upward combined force. Therefore, the hardening time is a very important indicator when PU is used to reinforce the soil. Too fast a solidification process results in an extremely short working time window for construction, whereas a very slow solidification time leads to slurry loss [13,34]. For the soil samples with PU contents of 12% and 16%, the strain change with depth was not significant because when there was more liquid PU among the soil particles, the overall cohesion was greater, and the residual amount was sufficient to bond the solid particles [35,36]. When the amount of slurry was low, grooves filled the surface of the particles. As the PU slurry content increased, a thin film formed around the particles, and the contact positions of the particles adhered. As the PU slurry content increased further, many PU connection bridges formed, which significantly improved the overall mechanical strength of the soil [36].

**Figure 9:**
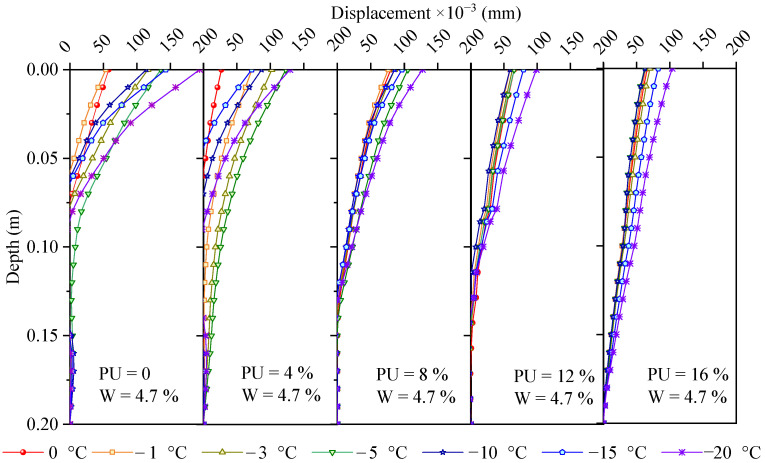
Displacement profiles of the soil samples with different PU contents at a water content of 4.7%.

The frost-heave displacement profile was obtained by integrating the strain profile shown in Figure 8 from the bottom to the top, as shown in Figure 9. Notably, the integral calculation of the frost-heave displacement must be from the bottom to the surface. When the ambient temperature is the lowest at −20 °C, the maximum displacements are 0.1, 0.1, 0.13, 0.12, and 0.2 mm for the soil samples with PU contents of 0%, 4%, 8%, 12%, and 16%, respectively. For the soil with a water content of 4.7%, the degree of frost heave is not large compared with that in previous reports because when the water content in the soil is lower than a critical value, water exists around the soil particles in the form of bound water and remains liquid after the temperature drops below 0 °C [37]. Even if a small amount of solid ice is present, it occupies the pore volume and does not change the spatial position of the solid particles. The critical value and maximum frost heave are affected by many factors, including shape, size, composition, pressure, and dry density [37].

**Figure 10:**
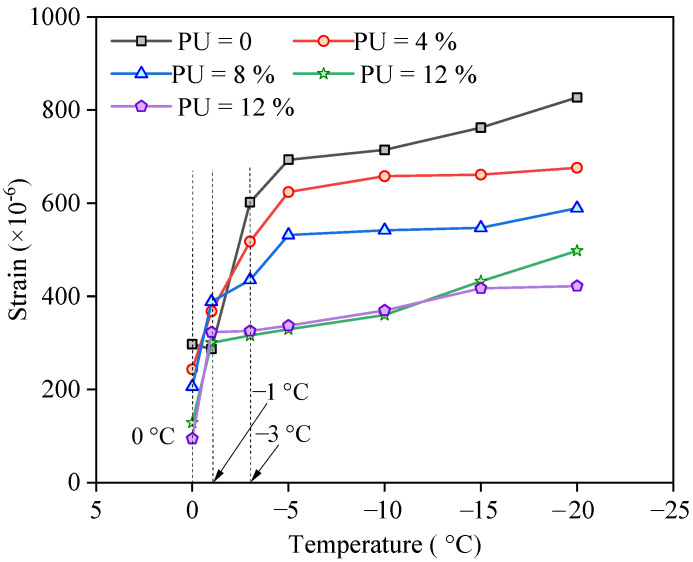
Average strain changes with temperature.

To further study the influence of the ambient temperature on frost heave, the average value of the strain profile data in Figure 8 was calculated, and the relationship between the average value and ambient temperature is shown in Figure 10. Twenty data points were used to calculate the average. When the soil temperature decreased below the freezing point, the liquid water gradually changed from liquid to ice. The freezing point is mainly determined by matric suction, which is affected by the pore size, chemical composition, and initial water content [38]. The higher the matric suction is, the lower the freezing point [38,39]. The influence of the freezing point on the phase change occurs in the temperature range from −8 to 0 °C, as reported in reference [40]. Figure 9 shows that freezing mainly occurs in the range of 0–5 °C. The higher the PU content is, the lower the frost-heave rate. The freezing point and frost-heave rate decreased after the addition of PU because the connection strength of the solid particles increased, and the void volume decreased, which resulted in an increase in matric suction. Chen et al. [41] stated that unsaturated soil freezing is different from saturated soil, which experiences frost shrinkage that may lead to settlement. The strain profiles are determined by water–ice phase change and the increase in matric suction [41]. In addition, the matric suction can change the water movement channels. Wang et al. [42] proposed a water–heat–vapor coupling model with consideration of the matric suction for the estimation of frost.

Taking the lowest temperature of −20 °C as the extreme cold weather condition, the effects of different PU contents on frost-heave strain and surface frost-heave displacement were calculated, as shown in Table 3. The calculation process is shown in Equations:

(9) $$\varepsilon_{p}=\frac{\varepsilon_{\mathrm{PU}(0)}-\varepsilon_{\mathrm{PU}(i)}}{\varepsilon_{\mathrm{PU}(0)}}\times 100\%,$$

(10) $$s_{p}=\frac{s_{\mathrm{PU}(0)}-s_{\mathrm{PU}(i)}}{s_{\mathrm{PU}(0)}}\times 100\%,$$

where $\varepsilon_{p}$ and $s_{p}$ are the maximum strain and displacement reduction percentage after PU reinforcement (%). $\varepsilon_{\mathrm{PU}(0)}$ and $\varepsilon_{\mathrm{PU}(i)}$ are the strains before and after grouting. $s_{\mathrm{PU}(0)}$ and $s_{\mathrm{PU}(i)}$ are the displacements before and after grouting (mm).

### 3.3. Influence of Adding Water on Frost Heave in PU-Reinforced Soil

**Figure 11:**
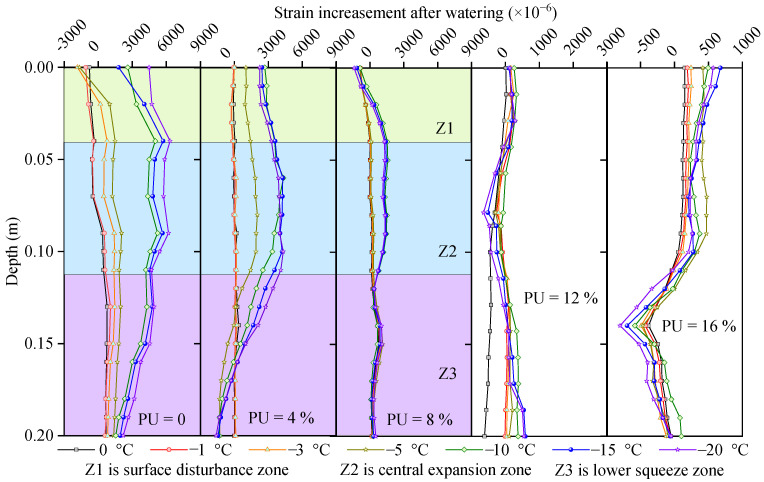
Strain difference between frost-heave samples with 10% and 4.7% moisture contents.

**Figure 12:**
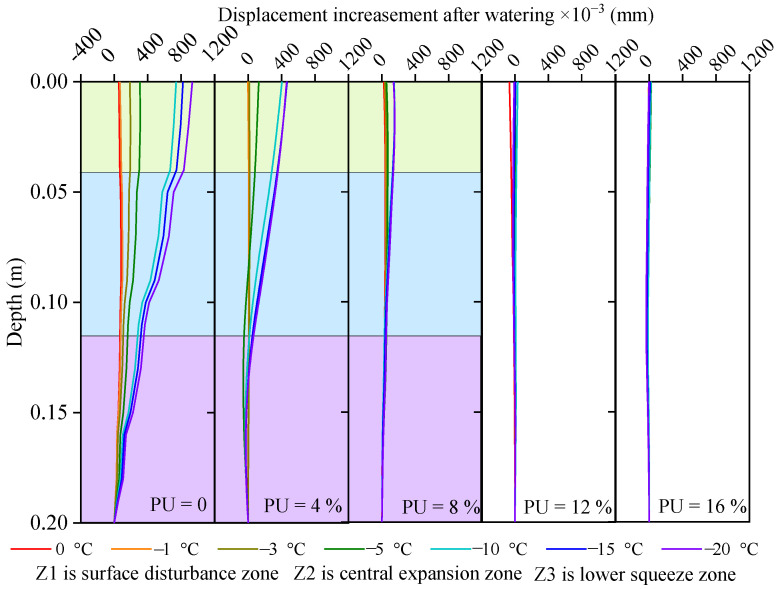
Displacement increasement between frost-heave samples with 10% and 4.7% moisture contents.

Figure 11 shows the strain increment (Δε), which is defined as the difference between the frost-heave amounts of the soil samples with 10% and 4.7% water contents. As the PU content increased, the effect of the water content gradually weakened. When the PU content was 12% or 16%, the added water had little effect on Δε, indicating that the soil particles were firmly bonded and that the frost-heave force was smaller than the bonding force. As the depth increased, the strain initially increased slightly before decreasing. It is smaller at the top because the surface-dripping process softens the soil sample and reduces the coupling between the SSC and the soil. The reason for the gradual decrease with increasing depth is that PU is enriched in the deep part under the action of gravity. The greater the depth, the more enriched it is, resulting in greater cohesion. In addition, as PU aggregates, the porosity of the soil decreases, making it difficult for water to penetrate, resulting in an uneven distribution of water in the soil and uneven frost heave. The displacement increment was obtained by integrating the strain in Figure 11, as shown in Figure 12.

For the soil samples with PU contents of 0%, 4%, and 8%, the depth can be divided into three zones according to the strain profiles: the surface disturbance zone (Z1), the central expansion zone (Z2), and the lower squeeze zone (Z3), respectively. The disturbance in Z1 refers to the destruction of the soil structure caused by watering. Notably, the strain reduction in Z1 indicates that the SSC fails to obtain the true deformation instead of the true soil strain. The frost heave of Z2 after the addition of water was the main reason for the frost-heave disaster of the entire frozen-soil roadbed. At any location, the soil is subjected to gravity, upper pressure, frost heave, and friction. Gravity and upper pressure were directed downward, frost heave was directed in all directions, and friction was in the direction opposite to the deformation. In Z2, the frost-heave force is greater than gravity and the overlying pressure; therefore, it manifests as expansion deformation. During the frost-heave process, Z2 exerts a downward pressure on Z3, resulting in compressive strain in Z3, as shown in Figure 11. Regarding the surface disturbance in Z1, Liu et al. [41] proposed a criterion for the interface coupling between SSC and surrounding soil and suggested using the elastic-plastic and softening models. During the process, the soil had two peak stress zones located in the upper and lower layers, which were consistent with the observation in Figure 11 and Figure 12. The coupling between SSC and soil can be divided into excellent, good, and general couplings [43]. Wu et al. [44] proposed that the compaction primarily occurs in the initial freezing stage (Five stages in total). The frost heave is caused by volume expansion, and the compression is driven by ice-lens formation and damage to soil structure [44].

The displacement profile in Figure 12 is obtained by integrating the calculation from the strain profile shown in Figure 11. Table 4 shows the growth rate of frost-heave displacement in soil reinforced with different PU contents as the moisture content increases from 4.7% to 10%. Because the water is unevenly distributed in the PU-reinforced soil after water is added, the maximum strain also exhibits spatial unevenness. Therefore, the increase in maximum strain will not be discussed quantitatively. When the moisture content increased from 4.7% to 10% and the ambient temperature was −20 °C, compared to the original reinforced soil, the frost-heave displacement of the reinforced soil with PU contents of 4%, 8%, 12%, and 16% increased by 49.34%, 14.93%, 7.48%, and 0.16%, respectively.

(11) $$s_{p}=\frac{\Delta s_{\mathrm{PU}(i)}}{\Delta s_{\mathrm{PU}(0)}}\times 100\%,$$

where $\Delta s_{\mathrm{PU}(i)}$ is the displacement increasement of the PU-reinforced soil after adding water (mm). $\Delta s_{\mathrm{PU}(0)}$ is the displacement increasement of the unreinforced soil (mm). *i* is the PU content.

It has been widely proven through laboratory tests that increasing the confining pressure reduces frost heave [45,46,47,48]. However, accurately measuring the in situ data of the confining pressure in the field is difficult. In this study, the strain profile monitored by the SSC was directly used to define the deformation zone. In addition to changing the mechanical equilibrium conditions, the confining pressure also affects frost heave by changing water migration; for example, the confining pressure can prevent water absorption and accumulation toward the freezing front [47]. Wang and Zhou [48] reviewed different frost-heave pressure models and emphasized the importance of water movement on pressure. Another important factor affecting the frost-heave pressure is the number of freeze–thaw cycles. The greater the number of cycles is, the smaller the frost-heave force, which eventually becomes stable. A decay coefficient was proposed, and a piecewise theoretical model was established to describe the attenuation process [45]. The combined effects of the ambient temperature, PU content, and watering on frost-heave are shown in Figure 12. To reduce the influence of friction at the cutter ring boundary, the frost-heave strain was considered the average value over the entire depth range.

**Figure 13:**
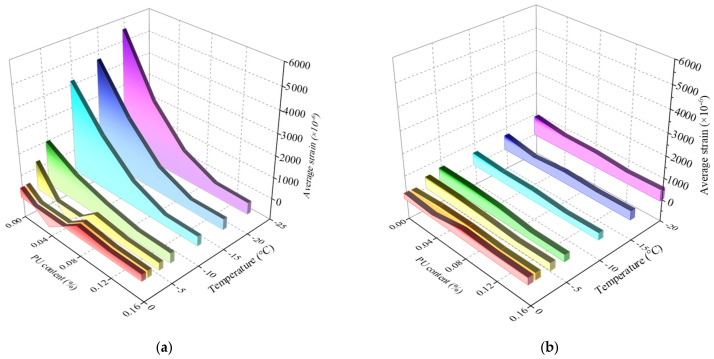
Influence of PU content and ambient temperature on frost heave, (**a**) after watering, and (**b**) before watering.

As shown in Figure 13, the factor that most significantly affected frost-heave was the PU content, followed by the water content. For soil with a natural water content of 4.7%, the influence of the ambient temperature and PU content can be neglected because ice cannot fully fill the pores during the freezing process. For the 10% water content sample, when the temperature was below −15 °C, the frost heave did not change with temperature, whereas in the range from −5 to −15 °C, the frost heave was sensitive to temperature. When the PU content was less than 8%, the frost-heave amount gradually decreased with increasing PU content, whereas when it was greater than 8%, the PU content had little effect on the frost-heave amount. Thus, the optimum PU content was determined to be 8%. Taking the market price of PU, 4000 USD per ton as the base, the material cost per cubic meter of soil is 145 $, 290 $, 435 $, and 580 $ for the soil with a PU content of 4%, 8%, 12%, and 16%, respectively.

## 4. Conclusions

OFDR technology was successfully used to monitor frost-heave strain profiles in PU-reinforced roadbed soil during laboratory tests. An SSC with acrylic plates was used to capture soil deformation. The influences of the PU, water, and ambient contents on frost heave were analyzed based on the distributed monitored data. The following conclusions were drawn:

(i) OFDR must be recalibrated in negative-temperature environments. The relationship between the strain shift caused by the temperature change and temperature is linear above 0 °C and nonlinear below 0 °C. This nonlinear relationship can be described by a cubic function, with an *R*^2^ value of 1.

(ii) PU and water content significantly improved the mechanical properties of the soil and reduced frost heave. The optimal PU content was determined to be 8%. Too little waste results in poor improvement, whereas excessive waste causes waste. When the PU content is less than 8%, the more water there is, the greater the degree of frost heave. However, when the PU content exceeded 8%, the soil particles were firmly bonded together, and adding water had little effect on frost heave. When the moisture content is 4.7% and the ambient temperature is −20 °C, compared with the original reinforced soil, the frost-heave displacement decreased by 33.27%, 47.43%, 71.65%, and 72.77%, respectively, after reinforcement with PU contents of 4%, 8%, 12%, and 16%.

(iii) When the moisture content increased from 4.7% to 10% and the ambient temperature was −20 °C, compared to the original reinforced soil, the frost-heave displacement of the reinforced soil with PU contents of 4%, 8%, 12%, and 16% increased by 49.34%, 14.93%, 7.48%, and 0.16%, respectively. When the PU content was less than 4%, the reinforcement effect was insignificant.

Although OFDR has been used to accurately monitor the frost heave of a PU-reinforced frozen-soil roadbed, further research, such as the influence of freeze–thaw cycles, upper vehicle loads, and water migration along cracks, is still needed in the field. In addition, the durability of strain-sensing cables in the field requires further verification. For the construction of new highway embankments, the PU content can be precisely controlled. However, for the treatment of frost heave and thaw settlement of existing embankments, PU is usually injected directly at a fixed pressure, making it difficult to control its content and thus making it difficult to control costs.

## Figures and Tables

**Table 1:** Chemical composition of soil samples measured by XRF.

Component	SiO_2_	Al_2_O_3_	CaO	Fe_2_O_3_	K_2_O	MgO	Others
Percent (%)	74.63	9.74	7.03	2.99	2.55	1.44	1.62

**Table 2:** Fitted parameters of the relationship between strain shift and temperature change.

A		B		C		D	
Value	Standard Error	Value	Standard Error	Value	Standard Error	Value	Standard Error
−926.43	4.15	46.51	0.47	−0.23	0.02	0.01	0

**Table 3:** Reduction percentage in frost heave of PU-reinforced soil when the ambient temperature is −20 °C, and the water content is 4.7%.

PU = 4%		PU = 8%		PU = 12%		PU = 16%	
Strain Reduction	Displacement Reduction	Strain Reduction	Displacement Reduction	Strain Reduction	Displacement Reduction	Strain Reduction	Displacement Reduction
32.77%	33.27%	34.36%	47.43	49.10%	71.65%	46.61%	72.77%

**Table 4:** The increase in percentage in frost-heave displacement of soils with different PU contents as the moisture content increases from 4.7% to 10% when the ambient temperature is −20 °C.

PU Content	PU = 4%	PU = 8%	PU = 12%	PU = 16%
Frost-heave displacement increasement percent after watering	49.34%	14.93%	7.48%	0.16%

## Data Availability

The original contributions presented in this study are included in the article. Further inquiries can be directed to the corresponding author.

## Abbreviations

The following abbreviations are used in this manuscript:

EPS	Expanded polystyrene
FBG	Fiber Bragg grating
FOS	Fiber optic sensors
FWD	Falling Weight Deflectometer
GPR	Ground penetrating radar
OFDR	Optical frequency-domain reflectometry
PU	Polyurethane
SSC	Strain-sensing cable
XPS	Extruded polystyrene
